# Intrinsic and Extrinsic Motivation Among Adolescent Ten-Pin Bowlers in Kuala Lumpur, Malaysia

**DOI:** 10.1515/hukin-2015-0025

**Published:** 2015-04-07

**Authors:** Eng-Wah Teo, Selina Khoo, Rebecca Wong, Eng-Hoe Wee, Boon-Hooi Lim, Shabesan Sit Rengasamy

**Affiliations:** 1 Sports Centre, University of Malaya, 50603, Kuala Lumpur, Malaysia.; 2 Kolej Universiti Tunku Abdul Rahman, 53300, Kuala Lumpur, Malaysia.; 3 Faculty of Education, University of Malaya, 50603, Kuala Lumpur, Malaysia.

**Keywords:** bowling, bowler type, gender, Self-Determination Theory, Sport Motivation Scale

## Abstract

Motivation has long been associated with sports engagement. However, to date no research has been performed to understand the domain of motivation among ten-pin bowlers. The purpose of this study was to investigate different types of motivation (i.e., intrinsic vs. extrinsic) based on self-determination theory from the perspective of gender and the bowler type (competitive vs. casual). A total of 240 bowlers (104 male, 136 female; 152 competitive, 88 casual) with a mean age of 16.61 ± 0.78 years were recruited in Kuala Lumpur. The Sport Motivation Scale, a 28-item self-report questionnaire measuring seven subscales (i.e., intrinsic motivation to know, intrinsic motivation to accomplish, intrinsic motivation to experience stimulation, extrinsic motivation to identify regulation, extrinsic motivation for introjection regulation, extrinsic motivation to external regulation, and amotivation) was administered. Results showed significant differences (t=10.43, df=239, p=0.01) between total scores of intrinsic and extrinsic motivation among ten-pin bowlers. There were significant gender differences with respect to intrinsic motivation to know, intrinsic motivation to accomplish, intrinsic motivation to experience stimulation, and extrinsic motivation to identify regulation. However, no significant bowler type differences were found for either the intrinsic (t=−1.15, df=238, p=0.25) or extrinsic (t=−0.51, df=238, p=0.61) motivation dimensions. In conclusion, our study demonstrated substantial intrinsic motivation for gender effects, but no bowler type effects among adolescent ten-pin bowlers.

## Introduction

The motivation to participate in sports is a complex phenomenon and is often associated with multiple engagement motives and different types of motivation (Pelletier et al., 2013). Understanding of motivation in sports engagement is indispensable because it is a vital predictor of ongoing and possible future sports commitment (Iso-Ahola and St. Clair, 2000). Therefore, a systematic examination of how different types of motivation (i.e., intrinsic vs. extrinsic) are associated with sports engagement is imperative for sustaining interest in sports.

**Figure 1:**
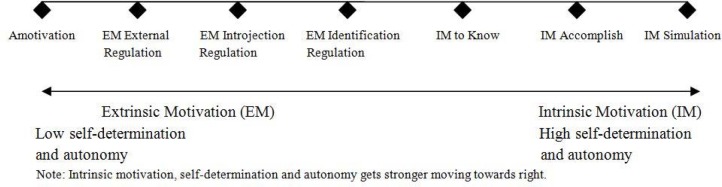
Self-determination continuum (Deci and Ryan, 1985; 2000)

Numerous sports studies have incorporated the self-determination theory (SDT) framework to understand sports engagement (Pelletier et al., 2013). SDT posits that individuals persistently attempt to control their own social environments to master three basic psychological needs, namely, the perceptions of competence, relatedness, and autonomy (Mallet, 2007). The perception of competence refers to the opportunity to express one’s own competence or capability (e.g., physical or mental prowess), whereas relatedness is the sense of belonging to a certain group or community. Autonomy is related to the ability to act or express oneself in ways that match one’s own needs and value system (Deci and Ryan, 1985). Autonomy is positively associated with self-determination and placed on an internalized continuum with amotivation (i.e., the least amount of self-determination) at one end (Figure 1), extrinsic motivation in the middle, and intrinsic motivation for stimulation at the other end (Ryan et al., 1992; Vallerand, 1997).

SDT posits that sports participation motivation is multidimensional, incorporating intrinsic, extrinsic, and amotivation (Deci and Ryan, 1985; Vallerand, 1997). Intrinsic motivation (IM) refers to participation exclusively for pleasure, fun, or satisfaction derived directly from the activity itself (Deci and Ryan, 1985). An intrinsically motivated individual will voluntarily participate in an activity in the absence of material rewards or external gain (Deci and Ryan, 1985). There are three subscales under the IM dimension, namely, IM to know, IM to accomplish, and IM to experience stimulation (Figure 1). IM to know includes constructs such as motivation to explore, learn, and understand. IM to accomplish refers to motivation to master, increase efficacy, and feel competent. Accomplishment-oriented individuals interact with the environment to feel competence, a sense of mastery, and satisfaction (Deci and Ryan, 1985). IM to experience stimulation refers to motivation to experience stimulating sensations (e.g., ecstasy, thrills) derived from participating in a given activity, such as bungee jumping or sky diving.

In contrast, extrinsic motivation (EM) is associated with an external locus of causality (Mallet, 2003) or “engagement as a means to an end and not for the sake of the activity itself” (Deci, 1975, p. 23). It was originally believed that EM was prompted purely by external contingencies (e.g., rewards); however, Ryan et al. (1992) argued that there are in fact different EM subscales that can be ordered along the self-determination continuum, such as EM for external regulation, EM for introjection, and EM for identification (Deci and Ryan, 1985).

EM for external regulation refers to behavior prompted by external motivation sources, such as material rewards (e.g., money, gifts, or rewards) or constraints imposed by others (e.g., criticisms or punishment). EM for introjection refers to external motivation (internal pressures) such as self-guilt to perform or anxiety. For example, athletes feel compel to succeed and be extremely fit but feel embarrassed when they do not live up to expectations (Pelletier et al., 1995). EM for identification occurs when individuals acknowledge and identify a certain behavior as significant and, therefore, perform it for extrinsic reasons such as to achieve personal goals. However, EM for identification is believed to be internally regulated and self-determined. For example, athletes participate in team-building sessions that could potentially contribute to their personal growth and development as athletes. Finally, amotivation, the lowest form of motivation, is located at the end of the SDT continuum; it is comparable to the concept of “learned helplessness” (Abraham et al., 1978, p. 50). SDT proposes that amotivated athletes may no longer identify any good reasons to train or play and may eventually disengage from sports.

Literature on sports motivation based on gender differences was inconclusive. Various studies revealed that female athletes demonstrated greater interest in fun, satisfaction, and pleasure, which are fundamental characteristics of IM (Chantal et al., 1996; Fortier et al., 1995; Pelletier et al., 1995). For example, the study by Gill et al. (1983) on children age 8–18 years old during a summer sports camp and Fortier et al. (1995) on 399 Canadian athletes found that male showed higher EM, while female were more IM oriented. Studies on 63 Bulgarian athletes (Chantal et al., 1996) and 365 US swimmers (Gould et al., 1985) found that male athletes demonstrated higher EM characterized by their focus on competition, winning, rewards, and recognition. A study by Sarrazin et al. (2002) of 335 French handball players revealed that female athletes were more IM oriented. In different study of 172 US collegiate student athletes, Kingston et al. (2006) found that male athletes with sports scholarships demonstrated significantly higher levels of EM, specifically EM for external regulation (obtaining measurable rewards), compared with females athletes. Similarly, Monazami et al. (2012) study’s which comprised of 112 Iranian athletes, revealed that male athletes scored higher in EM to identify regulation, EM to external regulation, and total EM compared with their counterparts. In contrast, Kim et al.’s (2003) study on 101 US and 298 Korean athletes found that IM was more prominent among males. Similarly, Zahariadis et al.’s (2005) study in Spain on 452 students found male students in PE classes exhibited higher IM than female ones. Finally, a recent study on 632 Malaysian athletes in track and field, found male athletes to be highly intrinsic motivated compare to their counterparts (Chin et al., 2012).

Beyond gender differences, comparative studies between athletes (competitive) and non-athletes (casual) have also generated substantial interest among sports psychologists. Literature revealed that competitive Greek rhythmic gymnasts (Koumpoula et al., 2011) and English soccer players (Lowther et al., 2004) exhibited higher IM and lower EM. Koumpoula et al. (2011) found that elite gymnasts showed high levels of task orientation, which is positively associated with greater IM. In contrast, casual players demonstrated higher EM and lower IM. In a separate study by Lowther et al. (2004) of 53 soccer players (professional, semiprofessional, and amateur) it was revealed that amateur players showed the lowest IM among the three groups and that professional players scored highly in IM but had low EM scores. Similarly, Koumpoula et al. (2011) studied 98 rhythmic gymnasts and found that gymnasts in the non-competitive group showed higher levels of EM for introjection regulation compared with those in the competitive group.

In conjunction with the above studies, sports engagement is hypothesized to be affected by demographic characteristics (i.e., gender) and by athletes’ ability levels. For example females were shown to favor IM, in contrary males were more EM oriented. Competitive athletes exhibited higher IM over EM as compared to their counterparts. The present study provides important information to sports stakeholders (e.g., sports psychologists, policy makers, tournament organizers, and coaches) whereby ten-pin bowling is one of the core sports in Malaysia. In the past Malaysian ten-pin bowlers have been successful in major international competitions, however, success is unsustainable leading to demotivation and even amotivation. Hence, to address these problems and attract youth’s interest, current knowledge of their motives and motivation is necessary. The finding could impact sports psychologists and coaches’ in making “informed” motivational choices (IM vs EM) that could determine sports performance. These findings are also invaluable for future sports development programs, sustainability of the sports itself, and laying the foundation for future cross-cultural comparison studies. Furthermore, to our knowledge, no study has investigated the motivation types of ten-pin bowlers globally nor locally. The purpose of this study was to investigate different types of motivation among ten-pin bowlers based on gender and the bowler type (i.e., competitive vs. casual).

## Material and Methods

### Participants

A total of 240 bowlers (104 males, 136 females) aged between 16 and 18 years (M=16.62 ±0.78 years) from Kuala Lumpur were recruited for the study. Participants, who were high school students with a minimum of 12 months of bowling experience, were categorized into two bowler types: competitive (n=152, M=16.52 ± 0.74 years) and casual (n=88, M=16.76 ± 0.83 years). Competitive bowlers were defined as bowlers who had competed at either the inter district, interstate, national, or international level. Casual bowlers were bowlers who played recreationally. The competitive bowlers were categorized into the following different levels: district (n=127), state (n=13), national (n=8), and international (n=4). Most participants identified themselves as Malay (66.7%), followed by Chinese (21.6%), Indian (9.6%), and others (2.1%). We also found that 74.6% of the participants’ parents were non athletes/women, 15.4% were ex-athletes/women, and 10% were still active in sports at the time of the study.

### Measures

The English version of the Sport Motivation Scale (SMS) developed by Pelletier et al. (1995) was administered. The 28-item SMS consists of seven factor subscales (i.e., IM to know, IM to accomplish, IM to experience stimulation, EM for identify regulation, EM for introjection regulation, EM for external regulation, and amotivation), with each subscale composed by four items. Each item was rated on a 7-point Likert-type scale ranging from 1 (does not correspond at all) to 7 (corresponds exactly). Higher scores indicate a higher level of motivation with a total score of 28 points being the maximum for each subscale. All subscales showed satisfactory internal consistency (α = 0.74–0.80), with a mean alpha of 0.75 (Pelletier et al., 1995). Martens and Webber (2002) reconfirmed the internal consistency of the SMS in a later study (α=0.70–0.82). Additional, validation studies were conducted by Li and Harmer (1996) using structural equation modeling (SEM) and by Jackson et al. (1998) for scale construct reliability and validity. Validation in different populations (Chatzisarantis et al., 2003; Zahariadis et al., 2005) also supported the internal consistency, construct validity, and simplex-like pattern of the SMS. A separate six-item demographic questionnaire was included to obtain the information about participants: race, age, gender, bowler type (i.e., competitive vs. casual), level of participation (i.e., district, state, national, international), and parents’ sports history.

### Procedure

Competitive bowlers were randomly recruited during the interschool bowling tournament in Kuala Lumpur, whereas casual bowlers were randomly recruited from 12 recreational school bowling clubs around Kuala Lumpur. Bowlers who had represented their respective schools, districts, states, or country were excluded from the casual bowler group. Research approval was obtained from the University of Malaya Ethics Committee. Participation was voluntary, and informed consent was obtained prior to the administration of the SMS questionnaire. Participants were briefed on the objectives and their ethical rights and were reminded that they were not obliged to respond to any of the questions if they were not comfortable doing so. Participants were free to withdraw at any time and were told that their withdrawal would not result in any penalty.

### Statistical Analysis

The IBM Statistical Package for the Social Sciences, SPSS Inc., Chicago Ill version 20.0 was used for data analysis. Data were checked for normality based on QQ plot, skewness, and kurtosis statistics. Gender differences (male vs. female) and bowler types (competitive vs. casual) were compared separately using independent t-tests for intrinsic and extrinsic motivation. The level of significance was set at alpha p<0.05. Effect size (ES) was later computed and categorized based on Cohen’s (1988) recommendations.

## Results

In general, data analysis revealed that ten-pin bowlers were more motivated by IM (M=4.89 ± 1.07) than by EM (M=4.32 ±1.03), with a significant difference at *p*=0.00 and a medium effect size (ES) of d=0.54.

### Gender

Table 1 showed that there was a significant gender difference in the IM dimension. Male bowlers (M=5.16 ± 0.97) showed higher total IM scores compared with female bowlers (M=4.69 ± 1.09; *p*=0.00, d=0.46). There were also significant gender differences in the three IM subscales: IM to know (M=5.29 vs. 4.63; *p*=0.00), IM to accomplish (M=5.21 vs. 4.80; *p*=0.01), and IM for stimulation (M=4.98 vs. 4.64; *p*=0.03).

Analysis of the EM dimensions revealed a significant gender difference. Male bowlers (M=4.53 ± 0.97) showed higher total EM scores compared with female bowlers (M=4.16 ± 1.04; *p*=0.01, d=0.37). There was also a significant gender difference in EM for identified regulation (M=5.18 vs. 4.64; *p*=0.00). However, no significant differences were observed in EM for external regulation (M=3.82 vs. 3.48; *p*=0.06) and EM for introjection regulation (M=4.59 vs. 4.39; *p*=0.14).

More comprehensive item-by-item analyses showed SMS scores (Table 2) ranging from 2.26–5.50. Item 8 (IM to accomplish; “Because I feel a lot of personal satisfaction while mastering certain difficult training techniques”) was scored the highest by male bowlers (M=5.50 ± 1.45), while other popular participation motives were from the IM dimension (items 8, 27, 15, and 1) and EM dimension (item 11). In contrast, female bowlers favored item 15 (IM to accomplish; “For the satisfaction I experience while perfecting my abilities”) (M=5.04 ± 1.40), with the most participation motives from the IM (items 1, 4, and 15) and EM (items 9 and 11) dimensions. Both genders chose item 19 (amotivation; “It is not clear to me anymore; I don’t really think my place is in sports”) as the least favored item (Male: M=2.26 ± 1.77; Female: M=2.82 ± 1.89).

### Bowler types

Analyses of bowler types along the IM dimension total scores revealed no significant difference between the two groups at *p*=0.25 (Table 1); however, the IM mean for competitive bowlers (M=4.95 ± 1.06) was slightly higher than that for casual bowlers (M=4.79 ± 1.07), with a small effect size of 0.15. There were no significant differences between bowler types in all IM subscales i.e., IM to know, IM to accomplish, and IM for stimulation (Table 1).

Analyses of bowler types along the EM dimension total scores also revealed no significant difference between groups at *p*=0.51 (Table 1). Competitive bowlers (M=4.35 ± 0.99) showed a slightly higher EM mean compared with casual bowlers (M=4.28 ± 1.08), with a very small effect size of 0.07. There were also no significant differences between bowler types in all EM subscales (Table 1).

Detailed item analyses of SMS scores based on the bowler type (Table 3) showed mean scores ranging from 2.51–5.48. Similar scoring patterns were observed between the competitive and casual bowlers. Competitive bowlers favored item 8 (M=5.26 ± 1.57), with the highest participation motives scores from the IM dimension (items 1, 8, 15, and 27) and the EM dimension (item 11). Casual bowlers, however, favored item 15 (M=5.18 ± 1.42), with the highest participation motives scores from the IM dimension (items 1, 4, and 15) and EM dimension (items 7 and 9). Again, item 19 was the least favored by both bowler groups (competitive: M=2.51 ± 1.84; casual: M=2.69 ± 1.89).

## Discussion

The purpose of this study was to investigate different types of motivation among ten-pin bowlers based on gender and the bowler type (i.e., competitive vs. casual). First, regardless of gender or the bowler type, the results revealed that ten-pin bowlers were significantly more motivated by IM. According to Standage et al. (2003), higher IM compared with EM is a positive sign because it reflects “ideal” engagement motives associated with sheer fun and fulfillment (Chatzisarantis et al., 2003). Ryan et al. (1992) hypothesized that high IM, especially in relation to being motivated by fun (IM for stimulation), mastery of skills (IM to accomplish), and self-improvement (IM to know), is a strong indicator of prolonged engagement in sports (Fox and Biddle, 1991; Gill et al., 1983).

Second, our study revealed evidence of a significant gender difference, with male bowlers recording higher IM scores than females. These results are in accordance with the study by Kim et al. (2003) on American versus Korean athletes, as well as with the study by Zahariadis et al. (2005) on Spanish PE students. Chin et al. (2012) also reported higher IM among Malaysian male track and field athletes as compared to female athletes. A plausible interpretation of these results may be related to early survival instincts toward being competent, which are relatively more dominant in males. To increase self-competence, males learned new techniques, strategies, and skills to become successful hunters (Ashford et al., 1993), which is related to the IM subscales of IM to know and IM to accomplish. In addition, it is also possible that in many parts of Asia, sports are still regarded as a predominantly male domain. Social etiquette, religious beliefs, and cultural norms require females to be feminine, friendly, and health- and beauty-conscious, which translates to a reduced need to be physically competent (Gill et al., 1983; Klint and Weiss, 1986). However, it should also be noted that earlier studies showed that female athletes exhibited higher IM than their male counterparts (Chantal et al., 1996; Fortier et al., 1995; Pelletier et al., 1995).

These anomalous findings could reflect the paradigm shift between modern-day and former (i.e., two decades ago) athletes.

Third, there were overall significant gender differences in the EM dimension; specifically, male bowlers recorded higher mean EM scores compared with female bowlers, corroborating previous studies by Chin et al. (2012), Fortier et al. (1995), and Gill et al. (1983). Our study confirmed the results of earlier studies that male athletes were more goal-oriented and prioritized rewards, medals, and fame compared with female athletes (Chantal et al., 1996; Chin et al., 2012; Kingston et al., 2006; Sarrazin et al., 2002). However, significant gender difference was observed in EM for identification, the closest EM subscale to the IM domain (based on self-determination continuum) similar to that reported by Monazami et al. (2012).

Fourth, our analyses revealed no significant difference between competitive (athletes) and casual (non-athletes) bowlers in the IM dimension. Logically, the criteria for bowler categorization (competitive vs. casual) are based on bowlers’ abilities, with little correlation with their motivation to participate. Therefore, no significant difference in the IM dimension between the groups was expected. Another plausible reason for this lack of difference is that young bowlers in our study were still on the learning curve except for state and national players. As a result, IM to know, IM to accomplish, and IM for stimulation may still have been relevant in both groups. Although there were no significant IM differences between bowler types, the total mean IM scores of the competitive bowlers (athletes) were comparatively higher than those of the casual bowlers (non athletes), consistent with the results of previous studies by Koumpoula et al. (2011) and Lowther et al. (2004).

Finally, no significant difference between bowler types (competitive and casual bowlers) was observed in the EM dimension. Although the difference was insignificant, the mean total EM scores for competitive bowlers (athletes) were slightly higher than those for casual bowlers (non athletes), especially for the EM for identification, similar to the study by Fortier et al. (1995). This result may be because competitive bowlers anticipate higher rates of success, fame, and acknowledgement compared with casual bowlers. According to Weiss and Chaumenton (1992), when engagement motives shift from fun or pure enjoyment to achievement, the pressure for recognition, status, and acknowledgement will proportionately increase. According to SDT theory (Deci and Ryan, 1985), individuals continuously try to conquer their environments in search of self-competence. Support for this argument can be observed when bowlers (competitive and casual) continuously try to improve themselves or perform better than they did previously, reflecting the natural tendency to demonstrate self-competence.

The present study provides practical implications associated with the promotion of better sports engagement and sports sustainability by underpinning intrinsic motivation. Understanding motivation is vital especially for coaches and sports psychologists to ensure elite athletes are continuously motivated to perform at their best level in competitions. However, motivation is no less important to non-athletes, in creating an enjoyable sporting experience, so that their sporting interest can be nurtured and maintained. Secondly literature implied that females were more intrinsically motivated but the current study showed that younger Malaysian males were not different from their counterparts. Therefore, we advocate for sensitivity towards different gender and bowler types needs to prevent sports disengagement and/or improved performance.

Study limitations can be traced to the lack of generalizability because data collection was carried out only in Kuala Lumpur. Beside, the newer version of the Sport Motivation Scale (Pelletier et al., 2013) was not used for the current study because data was collected prior to the Pelletier’s suggestion. Future studies could consider nationwide cross-sectional and multi-sport sampling to ensure better generalizability. Additionally, examination of the interaction of personal (e.g., personality, needs, interests, and goals) and situational (e.g., coaching style, facilities attractiveness, and team win-loss record) factors could be invaluable for understanding motivation among bowlers. In conclusion, this study revealed that intrinsic motivation and extrinsic motivation are substantially different according to gender but not according to bowler types.

## Figures and Tables

**Table 1:** Intrinsic and extrinsic motivation according to gender and the bowler type

Subscales		Mean (M)	Std Dev. (SD)	*t*-test	*p*	Effect Size (d)
Motivation (Total)	Intrinsic	4.89	1.07	10.43	0.00^*^	0.54
	Extrinsic	4.32	1.03			

IM between gender	Male	5.16	0.97	3.53	0.00^*^	0.46
	Female	4.69	1.09			
IM to Know	Male	5.29	1.08	4.40	0.00^*^	0.57
	Female	4.63	1.25			
IM to Accomplish	Male	5.21	1.11	2.85	0.01^*^	0.37
	Female	4.80	1.11			
IM to Stimulate	Male	4.98	1.14	2.19	0.03^*^	0.28
	Female	4.64	1.26			

EM between gender	Male	4.53	0.97	2.78	0.01^*^	0.37
	Female	4.16	1.04			
EM Reg. External	Male	3.82	1.31	1.89	0.06	0.25
	Female	3.48	1.39			
EM Reg. Introjection	Male	4.59	1.22	1.47	0.14	0.17
	Female	4.39	1.17			
EM Reg. Identification	Male	5.18	1.17	3.55	0.00^*^	0.47
	Female	4.64	1.15			

IM between bowler types						
	Competitive	4.95	1.06	−1.15	0.25	0.15
	Casual	4.79	1.07			
IM to Know	Competitive	5.00	1.22	−1.48	0.14	0.20
	Casual	4.76	1.11			
IM to Accomplish	Competitive	5.08	1.13	−1.78	0.08	0.24
	Casual	4.81	1.24			
IM for Stimulation	Competitive	4.78	1.20	0.10	0.92	−0.02
	Casual	4.80				

EM between bowler types						
	Competitive	4.35	0.99	−0.51	0.61	0.07
	Casual	4.28	1.08			
EM Reg. External	Competitive	3.62	1.27	0.07	0.94	−0.01
	Casual	3.64	1.51			
EM Reg. Introjection	Competitive	4.48	1.18	−0.42	0.68	0.06
	Casual	4.41	1.23			
EM Reg. Identification	Competitive	4.93	1.22	−0.98	0.33	0.13
	Casual	4.78	1.13			

* Significant at p < 0.05; IM=intrinsic motivation; EM=extrinsic motivation

**Table 2:** Intrinsic and extrinsic motivation between genders

Item	Male		Female		Total	
Why do you practice sport?	M	SD	M	SD	M	SD
1. For the pleasure I feel living an exciting experience.	5.39	1.57	4.93	1.71	5.13	1.66
2. For the pleasure it gives me to know more about the sport that I practice.	5.16	1.51	4.60	1.84	4.85	1.73
3. I used to have good reasons for doing sport, but now I am asking myself whether I if I should continue doing it.	3.05	2.05	2.96	1.96	3.00	2.00
4. For the pleasure of discovering new training techniques.	5.27	1.54	4.85	1.53	5.03	1.55
5. I don’t know anymore; I have the impression of being incapable of succeeding in sports.	3.08	1.86	3.36	1.80	3.18	1.83
6. Because it allows me to be well regarded by people that I know.	4.26	1.86	3.59	1.79	3.88	1.85
7. Because, in my opinion, it is one of the best ways to meet people.	5.22	1.56	4.85	1.59	5.01	1.58
8. Because I feel a lot of personal satisfaction while mastering certain difficult training techniques.	5.50	1.45	4.85	1.61	5.13	1.57
9. Because it is absolutely necessary to do sports if one wants to be in shape.	5.09	1.76	4.96	1.71	5.01	1.73
10. For the prestige being an athlete.	3.96	1.87	3.51	1.75	3.71	1.81
11. Because it is one of the best ways I have chosen to develop other aspects of myself.	5.34	1.59	4.85	1.48	5.06	1.54
12. For the pleasure I feel while improving some of my weak points.	5.20	1.63	4.68	1.56	4.90	1.61
13. For the excitement I feel when I am really involved in the activity.	4.47	1.74	4.48	1.74	4.48	1.74
14. Because I must do sports to feel good myself.	4.38	1.78	4.59	1.62	4.50	1.69
15. For the satisfaction I experience while I am perfecting my abilities.	5.41	1.51	5.04	1.40	3.59	1.46
16. Because people around me think it is important to be in shape.	3.59	1.79	3.98	2.10	3.81	1.98
17. Because it is a good way to learn lots of things which could be useful to me in other area of my life.	5.11	1.62	4.68	1.47	4.86	1.55
18. For the intense emotions I feel doing a sport that I like.	4.85	1.58	4.66	1.69	4.74	1.64
19. It is not clear to me anymore; I don’t really think my place is in sports.	2.26	1.77	2.62	1.89	2.58	1.86
20. For the pleasure that I feel while executing certain difficult movements.	4.61	1.74	4.47	1.64	4.53	1.68
21. Because I would feel bad if I’m not taking time to do it	4.14	1.95	3.76	1.73	3.93	1.83
22. To show others how great I am at my sport.	3.46	2.04	2.85	1.82	3.12	1.94
23. For the pleasure I feel while learning new training techniques that I had never tried before.	5.27	1.57	4.67	1.56	4.93	1.59
24. Because it is one of the best ways to maintain good relationship with my friends.	5.18	1.67	4.37	1.74	4.72	1.76
25. Because I like being totally immersed in the activities.	5.21	1.61	4.49	1.80	4.80	1.75
26. Because I must do sports regularly.	4.74	1.83	4.13	1.71	4.39	1.79
27. For the pleasure of discovering new performance strategies.	5.46	1.28	4.39	1.71	4.85	1.63
28. I often ask myself; I can’t seem to achieve the goals that I set for myself.	4.04	1.92	4.00	1.81	4.02	1.85

**Table 3:** Intrinsic and extrinsic motivation between bowler types

Item	Male		Female		Total	
Why do you practice sport?	M	SD	M	SD	M	SD
1. For the pleasure I feel living an exciting experience.	5.21	1.63	4.93	1.71	5.13	1.66
2. For the pleasure it gives me to know more about the sport that I practice.	4.88	1.69	4.80	1.79	4.85	1.73
3. I used to have good reasons for doing sport, but now I am asking myself whether I if I should continue doing it.	2.90	1.99	3.17	2.01	3.00	2.00
4. For the pleasure of discovering new training techniques.	5.02	1.61	5.05	1.45	5.03	1.55
5. I don’t know anymore; I have the impression of being incapable of succeeding in sports.	3.20	1.82	3.16	1.86	3.18	1.83
6. Because it allows me to be well regarded by people that I know.	3.91	1.83	3.82	1.89	3.88	1.85
7. Because, in my opinion, it is one of the best ways to meet people.	4.96	1.62	5.09	1.54	5.01	1.58
8. Because I feel a lot of personal satisfaction while mastering certain difficult training techniques.	5.26	1.57	4.91	1.55	5.13	1.57
9. Because it is absolutely necessary to do sports if one wants to be in shape.	4.99	1.78	5.06	1.65	5.01	1.73
10. For the prestige being an athlete.	3.71	1.84	3.70	1.78	3.71	1.81
11. Because it is one of the best ways I have chosen to develop other aspects of myself.	5.12	1.55	4.97	1.53	5.06	1.54
12. For the pleasure I feel while improving some of my weak points.	5.03	1.64	4.69	1.55	4.90	1.61
13. For the excitement I feel when I am really involved in the activity.	4.47	1.74	4.48	1.74	4.48	1.74
14. Because I must do sports to feel good myself.	4.29	1.63	4.85	1.74	4.50	1.69
15. For the satisfaction I experience while I am perfecting my abilities.	5.22	1.48	5.18	1.42	5.20	1.46
16. Because people around me think it is important to be in shape.	3.74	1.85	3.93	2.19	3.81	1.98
17. Because it is a good way to learn lots of things which could be useful to me in other area of my life.	4.86	1.57	4.86	1.50	4.86	1.55
18. For the intense emotions I feel doing a sport that I like.	4.66	1.62	4.89	1.67	4.74	1.64
19. It is not clear to me anymore; I don’t really think my place is in sports.	2.51	1.84	2.69	1.89	2.58	1.86
20. For the pleasure that I feel while executing certain difficult movements.	4.72	1.62	4.19	1.75	4.53	1.68
21. Because I would feel bad if I’m not taking time to do it.	4.06	1.90	3.70	1.69	3.93	1.83
22. To show others how great I am at my sport.	3.13	1.89	3.09	2.03	3.12	1.94
23. For the pleasure I feel while learning new training techniques that I had never tried before.	5.04	1.64	4.74	1.50	4.93	1.59
24. Because it is one of the best ways to maintain good relationship with my friends.	4.88	1.77	4.45	1.72	4.72	1.76
25. Because I like being totally immersed in the activities.	4.86	1.79	4.70	1.70	4.80	1.75
26. Because I must do sports regularly.	4.59	1.71	4.05	1.88	4.39	1.79
27. For the pleasure of discovering new performance strategies.	5.08	1.54	4.47	1.71	4.85	1.63
28. I often ask myself; I can’t seem to achieve the goals that I set for myself.	3.93	1.88	4.17	1.80	4.02	1.85
